# Detecting potential mechanism of vitamin D in treating rheumatoid arthritis based on network pharmacology and molecular docking

**DOI:** 10.3389/fphar.2022.1047061

**Published:** 2022-11-30

**Authors:** Xiaoyu Xu, Hongyu Luo, Qian Chen, Zikang Wang, Xixuan Chen, Xiaping Li, Huan Chen, Miao Wang, Yingyue Xu, Min Dai, Jianwei Wang, Xuekuan Huang, Bin Wu, Yanping Li

**Affiliations:** ^1^ College of Traditional Chinese Medicine, Chongqing Medical University, Chongqing, China; ^2^ Department of Rheumatology, Chongqing Hospital of Traditional Chinese Medicine, Chongqing, China; ^3^ Chongqing Key Laboratory of Traditional Chinese Medicine for Prevention and Cure of Metabolic Diseases, College of Traditional Chinese Medicine, Chongqing Medical University, Chongqing, China

**Keywords:** vitamin D, rheumatoid arthritis, network pharmacology, molecular docking, diagnostic marker or target

## Abstract

**Aim:** Vitamin D plays a vital role in Rheumatoid arthritis (RA). However, the mechanism of vitamin D and rheumatism is still unclear. Therefore, a strategy based on network pharmacology and molecular docking was used to explore the mechanism of vitamin D and RA.

**Methods:** The targets of RA were obtained from the GeneCards database and Therapeutic Targets Database, and the targets of vitamin D were obtained from the Drugbank database and STITCH database. Next, overlapping genes were identified by Venny, and further Gene ontology (GO), Kyoto Encyclopedia of Genes and Genomes (KEGG), and molecular docking analyses were performed.

**Results:** A total of 1,139 targets of RA and 201 targets of vitamin D were obtained. A total of 76 overlapping genes were identified by Venny. The enrichment analysis showed that cell proliferation, immune response, and apoptotic process were the critical biological processes of vitamin D in treating RA. Antifolate resistance, osteoclast differentiation, and the nuclear factor-kappa B (NF-κB) signalling pathway are fundamental mechanisms of vitamin D in treating RA. According to further molecular docking, ALB, TNF, CASP3, and TP53 may be important punctuation points or diagnostic markers for future RA treatment.

**Conclusion:** By analysing overlapping genes of diseases and drugs, this study confirmed that ALB, TNF, CASP3, and TP53 may be essential markers or diagnostic markers for future RA treatment.

## Introduction

RA is an autoimmune disease characterised by chronic inflammation of the synovial joints, progressive bone erosion, and joint destruction. The prevalence of RA is 0.3%–1.5% in the world; the development of RA can lead to joint damage, disability, reduced quality of life, and multiorgan disease (Bueno et al., 2021; Fan et al., 2021; Maiuolo et al., 2021; Masoumi et al., 2021). The pathogenesis of RA is complex and still unclear, and studies have shown that it is closely related to genetics and immunity (Trenkmann et al., 2010) as the burden and disability of RA have increased worldwide (Ru et al., 2019; Mouterde et al., 2020; Hegarty et al., 2021). Due to the pathophysiological heterogeneity of RA, there are many clinical treatments, including antirheumatic drugs, glucocorticoids, non-steroidal anti-inflammatory drugs, inflammatory cytokine inhibitors, and methotrexate (MTX), but the prognosis is poor and there are various side effects (Nawaz et al., 2021; Yan et al., 2021; Zhao et al., 2021). Therefore, we must take some measures against RA. Although more biological agents have been used to improve patients’ quality of life, the treatment of rheumatoid arthritis still presents many challenges.

Vitamin D is a steroid hormone that regulates calcium homeostasis and skeletal health (Chang et al., 2021). Some evidence suggests that vitamin D is an immunomodulatory hormone with an established role in bone mineralisation (El-Banna and Gado, 2020; Verma et al., 2021). In a large-scale vitamin D and omega-3 trial (VITAL) that lasted approximately 5 years, the impact of supplementation with vitamins and omega-3 fatty acids on autoimmune disease rates, including rheumatoid arthritis, polymyalgia rheumatic, autoimmune thyroiditis and psoriatic arthritis, was reported. The results showed that 5 years of vitamin D supplements (with or without fatty acids) could reduce 22% of autoimmune diseases. For the vitamin D group, the treatment group included 123 participants. The placebo group included 155 participants who confirmed autoimmune diseases (hazard ratio 0.78%, 95% confidence interval 0.61 to 0.99, *p* = 0.05) (Hahn et al., 2022). A trial of 40 patients with active RA showed that vitamin D supplementation for 3 months can increase regulatory T cells, reduce Disease Activity Score-28 scores and modulate the immune system (El-Banna and Gado, 2020). Another study showed that vitamin D deficiency was associated with a 72.6% higher prevalence of new fractures in Japanese women over 50 with rheumatoid arthritis. Therefore, vitamin D plays a vital role in RA, providing an important addition to the previously available therapy options (Zhang et al., 2021). However, the mechanism between vitamin D and rheumatic diseases is still unclear. Because of the significant clinical effect, the study systematically predicts the potential mechanism of the molecular regulation of vitamin D on RA. Furthermore, it provides potential therapeutic targets for clinical and basic research.

Network pharmacology is an intelligent strategy to explore the complex mechanism of disease and drugs (Li et al., 2022). Molecular docking is a method based on computer modelling of structures. It is widely used to assist in the discovery of new medicines (Pinzi and Rastelli, 2019). Therefore, we designed this experiment to explore the mechanism of vitamin D and RA *via* network pharmacology combined with molecular docking.

## Materials and methods

### Work flow

**FIGURE 1:**
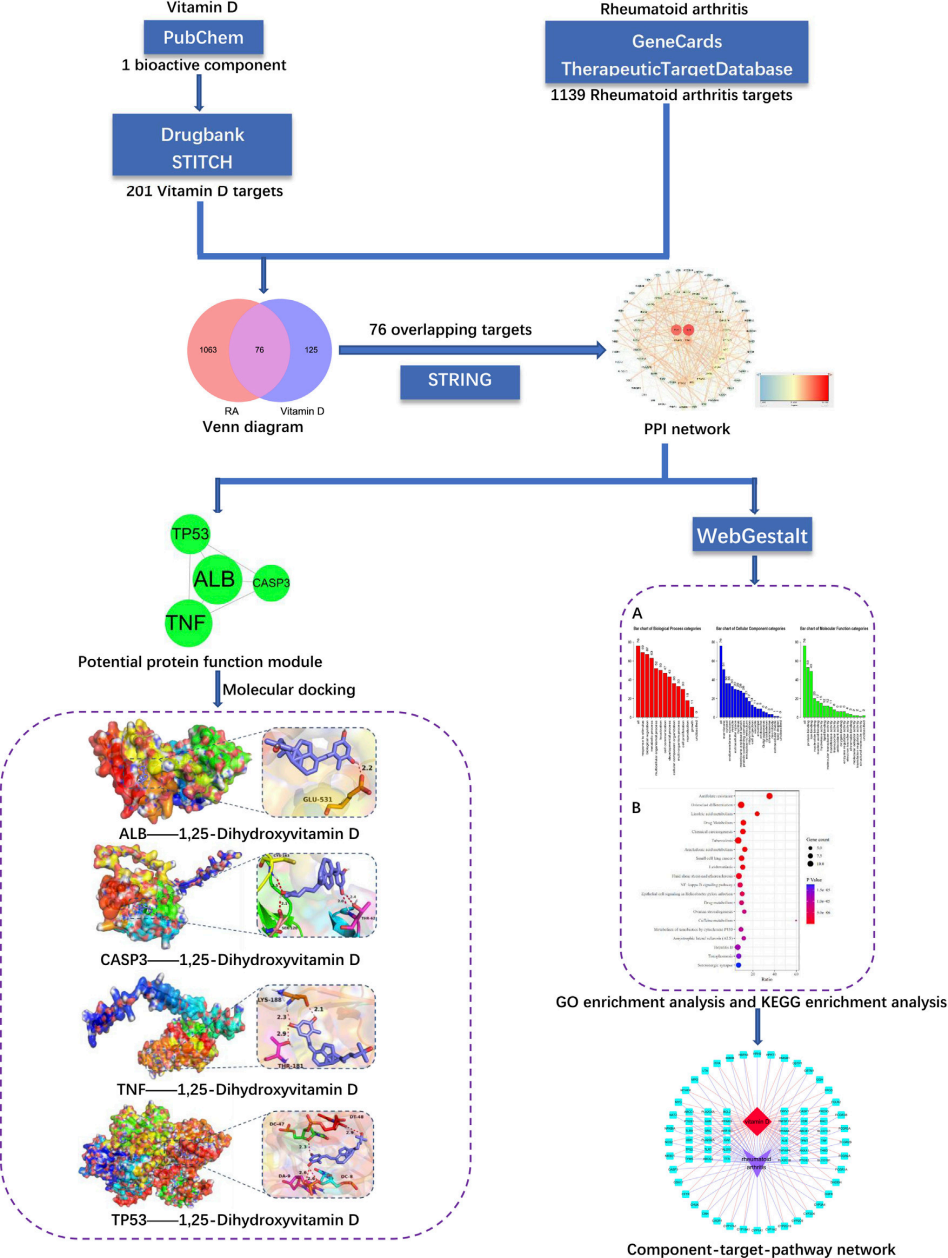
Schematic diagram of the study.

In this research, an intelligent method based on network pharmacology was designed, and the technique was divided into three steps. The first step was collecting overlapping RA and vitamin D genes from databases. The second step was to analyse the overlapping genes through enrichment analysis and protein-protein interaction (PPI) networks. The third step was to further analyse the correlation between vitamin D and RA through molecular docking. The process of this research is shown in Figure 1.

### Obtain overlapping genes

The genes related to RA were obtained from the GeneCards database (www.genecards.org/) (Safran et al., 2010) and Therapeutic Targets Database (TTD) (Wang et al., 2020) (db.idrblab.net/ttd/). The targets of vitamin D were obtained from the Drugbank database (www.drugbank.ca/) (Law et al., 2014) and the STITCH database (stitch.embl.de/) (Kuhn et al., 2010). Then, Venn platform (http://bioinformatics.psb.ugent.be/webtools/Venn/) was used to screen the overlapping targets of vitamin D and RA to determine the potential targets of vitamin D in the treatment of rheumatic diseases, and the “drug–compound–disease” network map of vitamin D against rheumatic diseases was made using Cytoscape software (Shannon et al., 2003; Pirooznia et al., 2007).

### Enrichment analysis

To analyse the mechanism related to RA and vitamin D systematically, GO enrichment analysis was performed. The top 20 biological processes were selected for functional annotation in the analysis. KEGG enrichment analysis was performed to investigate functional targets’ biological function in the pathogenesis of common targets (*p* < 0.05). The top 20 pathway terms were selected for functional annotation (*p* < 0.05). The enrichment analyses above were performed through DAVID Bioinformatics Resources 6.8 (david.ncifcrf.gov/) (Huang et al., 2009). The enrichment analysis results were visualised using the OmicShare cloud platform (www.omicshare.com/) (Zhang et al., 2019), and Cytoscape’s “drug–target–pathway” network map of vitamin D against RA was made software for further study.

### Construction of the protein-protein interaction network

The string database (string-db.org) was used to collect functional protein associations of common targets. The interaction data were imported into Cytoscape (v3.7.2) to establish a protein-protein interaction (PPI) network and obtain the genes with a higher correlation with common targets (Szklarczyk et al., 2019; Nogales et al., 2022). A network analyser, a plug-in of Cytoscape, was utilised to analyse the topological parameters of node degree in the PPI network. The node degree was defined as the number of edges associated with it. The targets with high degree values were identified as the core targets of common targets.

### Molecular docking

The 2D chemical structures of the main active components of vitamin D were obtained through the PubChem database (https://pubchem.ncbi.nlm.nih.gov/), and the relevant core targets were found in the PDB database (https://www.rcsb.org/). For the 3D structure of the protein, molecular docking was performed by AutoDock software to obtain the site with the highest binding energy and finally visualised by PyMOL software (Seeliger and De Groot, 2010).

## Results

### Information on target acquisition

**FIGURE 2:**
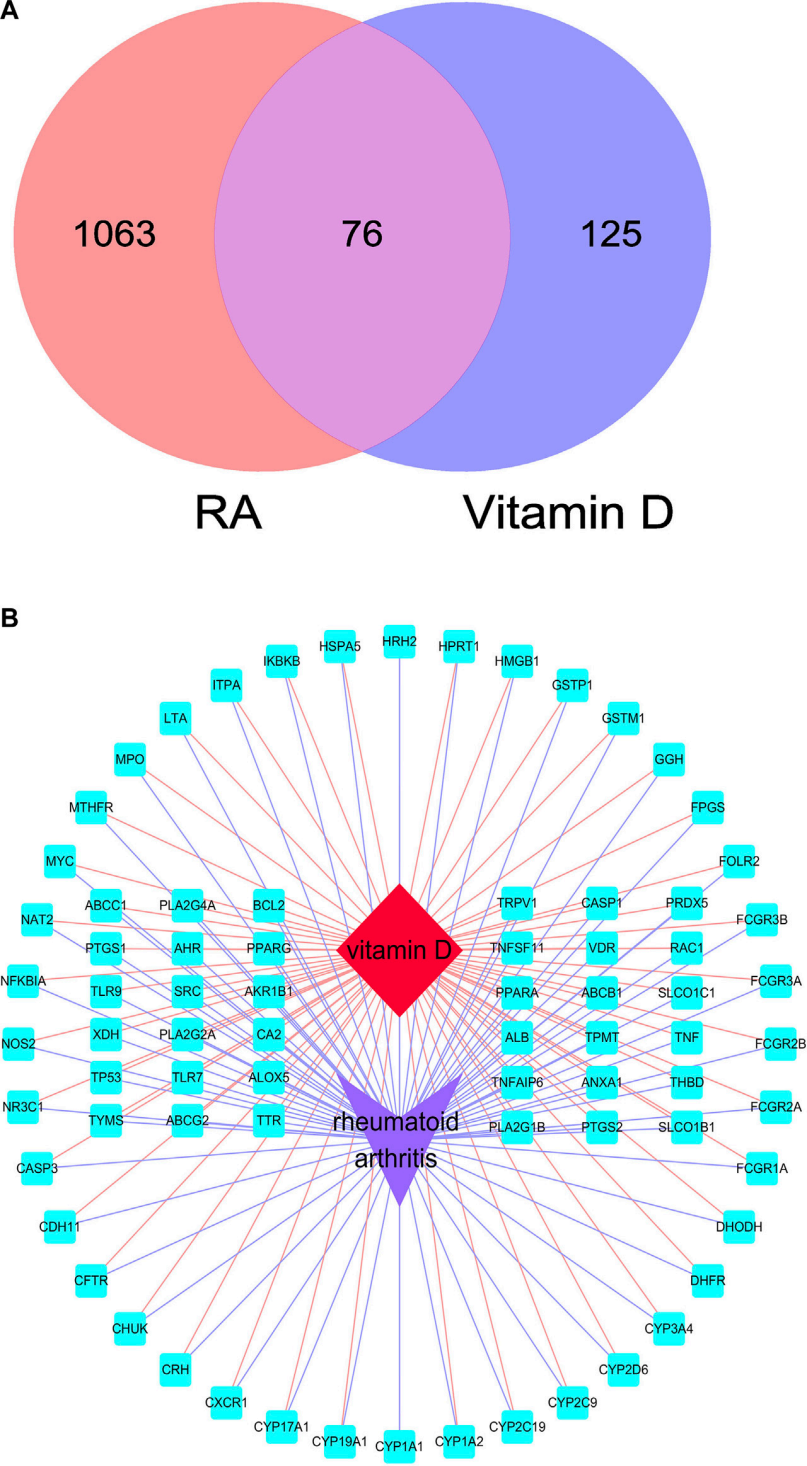
Overlapping targets of RA and vitamin D. **(A)** Overlapping targets identified by Venny. **(B)** “vitamin D–overlapping genes–RA” network map. The red diamond represents RA, the purple arrow represents vitamin D, and the blue square represents overlapping genes.

A total of 1,093 genes related to RA were collected from the GeneCards database according to the standard of having a relevance score more significant than the median. A total of 131 genes were obtained from the TTD database. After removing duplicate targets, 1,139 genes related to RA were identified after data collation (Supplementary Table S1). A total of 192 approved targets of vitamin D were acquired from the Drugbank database, and nine predicted targets were obtained from the STITCH database (Supplementary Table S2). After data collation, 201 targets of vitamin D were obtained by Venny (Figure 2A). There were 1,139 potential targets in RA which shared 76 common targets with vitamin D-related targets (Supplementary Table S3). Subsequently, the “vitamin D–overlapping genes–RA” network map was created by Cytoscape (Figure 2B).

### The construction of protein-protein interaction network

**FIGURE 3:**
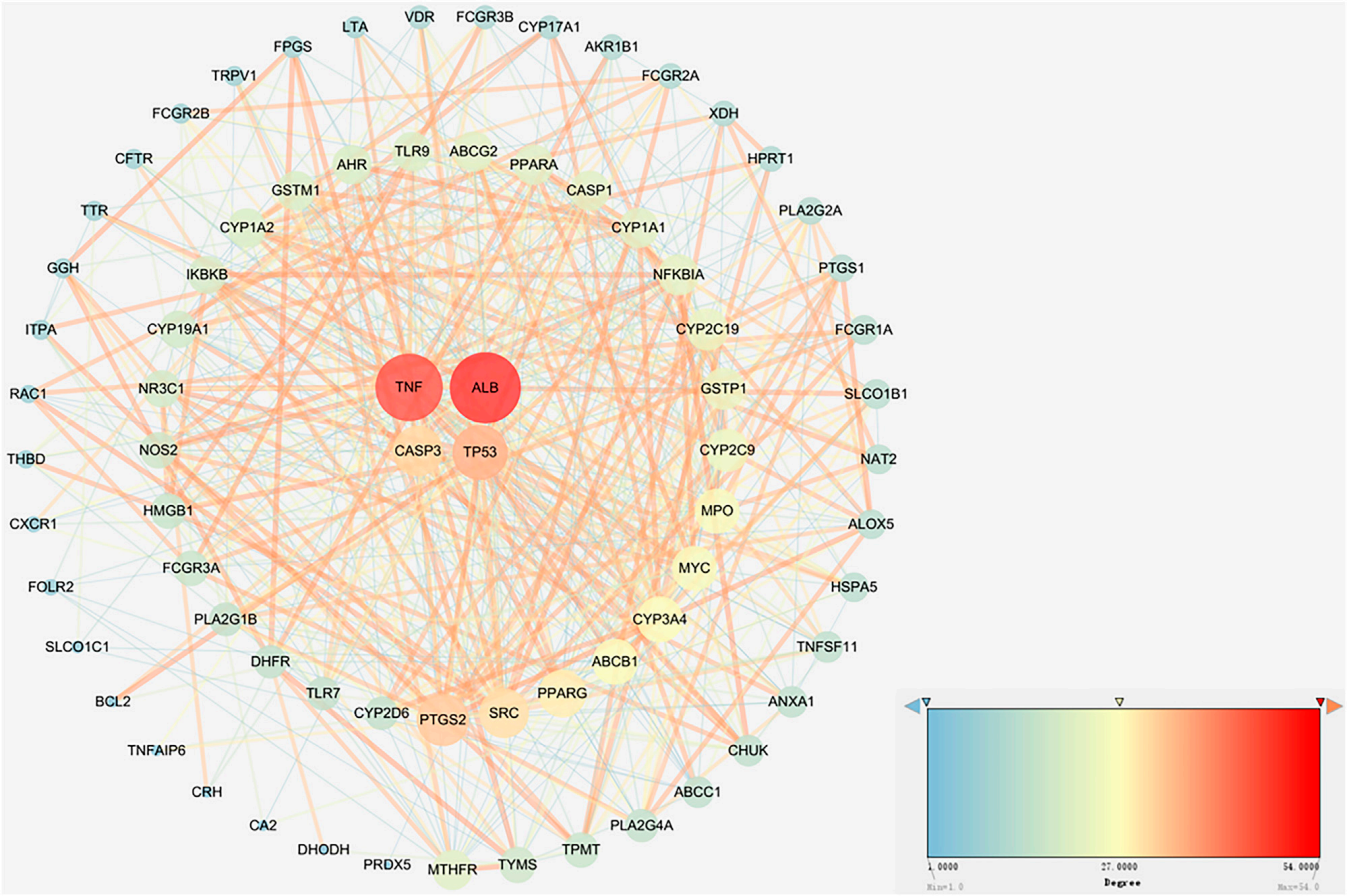
Network analysis of protein interactions. A circle represents an overlapping gene. The larger the node size and the redder the color, the higher the degree value.

**FIGURE 4:**
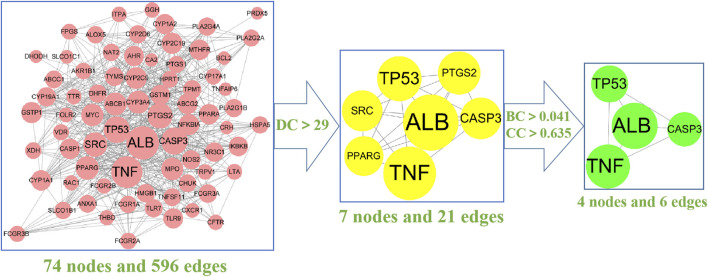
The screening process of the PPI network by Cytoscape: The 74 pink nodes represent the intersection targets of PPI network; The 7 yellow nodes refer to the target obtained after the first screening (DC > 29); The 4 green nodes are the final core targets obtained after the second screening (BC > 0.041, CC > 0.635). (degree (DC), betweenness centrality (BC), closeness centrality (CC).The larger the circle is, the more critical the target is.

To further analyse the relationship between RA and vitamin D, the Bisogenet program in Cytoscape software was used to analyse the PPI network map in the STRING database, as shown in Figure 3. The network comprises 74 potential targets and 596 edges (excluding free nodes CDH1 and HRH2). Among them, the key targets are as follows (Table 1). To further confirm our conclusion, the degree of freedom and betweenness of the topological parameters of the Cytoscape plug-in Network Analyse were used to evaluate the critical potential targets of vitamin D intervention in RA. For the first time, a Degree Centrality (DC) value more than twice the median was used as the screening index, and for the second time, Betweenness Centrality (BC) and Closeness Centrality (CC) greater than the median were used as the screening index, among which seven potential core targets for vitamin D intervention in RA were obtained. Through analysis, we finally identified four crucial targets: TNF, ALB, CASP3, and TP53 (DC > 29, BC > 0.041, and CC > 0.635). The results are shown in Figure 4 (Supplementary Table S4). Interestingly, the results are the same as for the ranking by degree, which proves that these four targets are indeed the key targets for vitamin D treatment of RA.

**TABLE 1:** The top 20 targets in PPI.

Gene symbol	The name of protein	Degree	Betweenness
ALB	Albumin	54	0.18151
TNF	Tumor necrosis factor	50	0.13549
TP53	Cellular tumor antigen p53	37	0.05277
CASP3	Caspase-3	34	0.03019
PTGS2	Prostaglandin G/H synthase 2	32	0.04058
SRC	Proto-oncogene tyrosine-protein kinase Src	31	0.02696
PPARG	Peroxisome proliferator-activated receptor gamma	29	0.01983
ABCB1	ATP-dependent translocase ABCB1	27	0.03690
CYP3A4	Cytochrome P450 3A4	27	0.02192
MYC	Myc proto-oncogene protein	26	0.02493
MPO	Myeloperoxidase	26	0.02685
CYP2C9	Cytochrome P450 2C9	24	0.01530
CYP2C19	Cytochrome P450 2C19	23	0.01313
GSTP1	Glutathione S-transferase P	23	0.01254
NFKBIA	NF-kappa-B inhibitor alpha	22	0.00508
ABCG2	Broad substrate specificity ATP-binding cassette transporter ABCG2	21	0.01931
MTHFR	Methylenetetrahydrofolate reductase	21	0.02910
CYP1A1	Cytochrome P450 1A1	21	0.00655
PPARA	Peroxisome proliferator-activated receptor alpha	21	0.00652
CASP1	Caspase-1	21	0.00699

### Information on GO enrichment and kyoto encyclopedia of genes and genomes analyses

**FIGURE 5:**
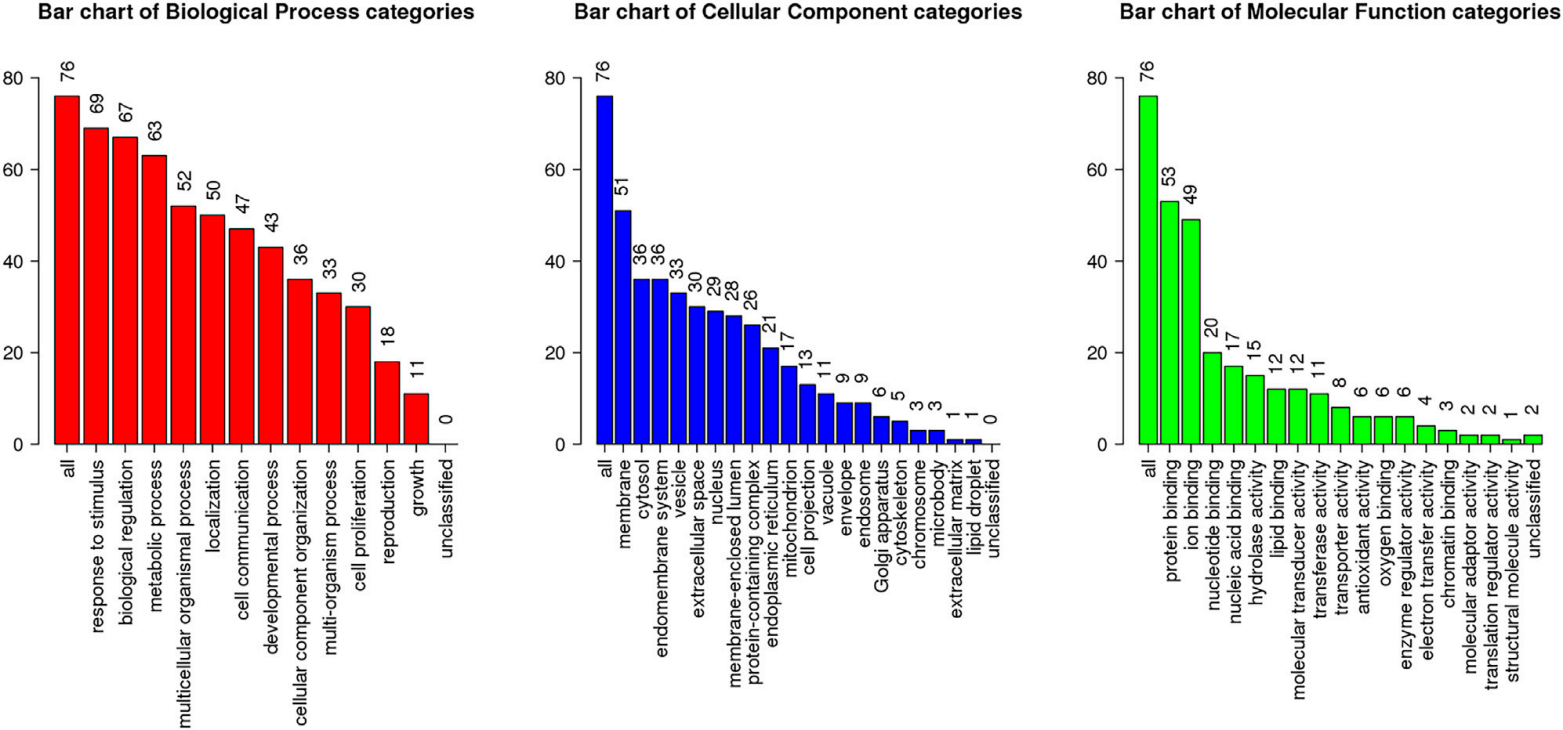
GO enrichment analysis of overlapping genes. The X-axis represents the GO enrichment entry, and the Y-axis represents the number of enriched genes in the GO enrichment terms, p < 0.05.

**FIGURE 6:**
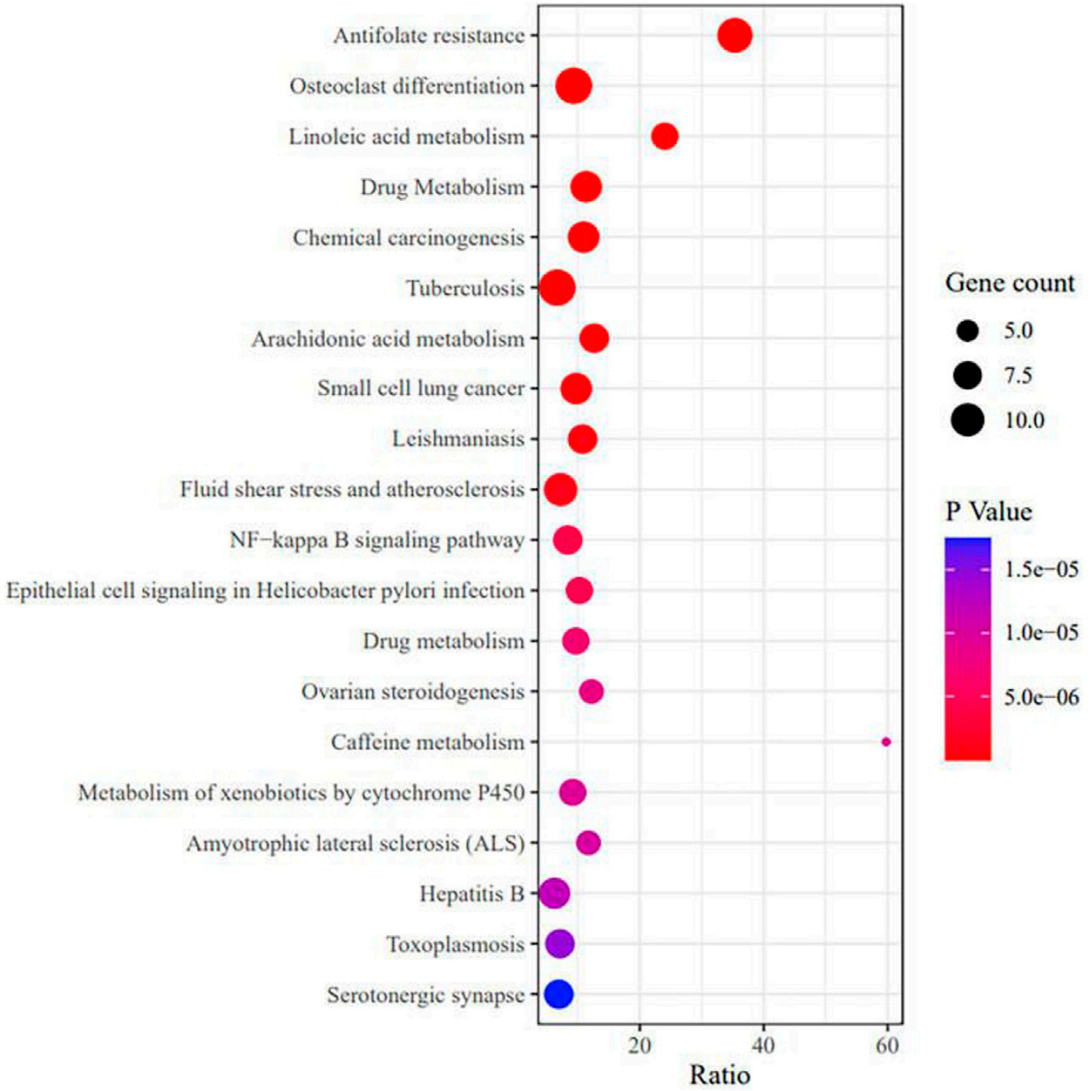
KEGG enrichment analysis of overlapping genes. The X-axis represents the gene enrichment rate, and the Y-axis represents the KEGG enrichment terms, p < 0.05.

**FIGURE 7:**
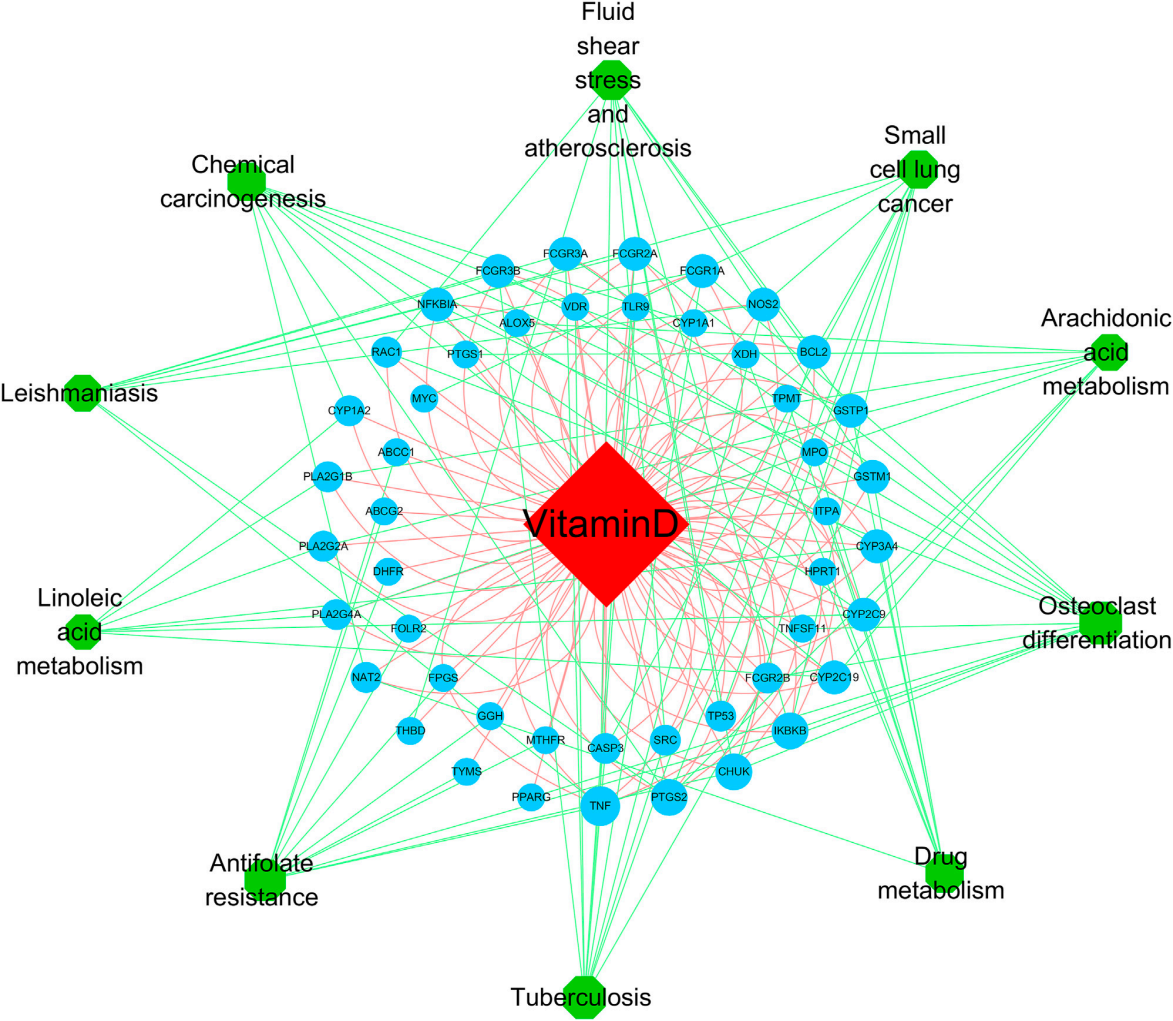
The vitamin D–target–pathway diagram of the top 10 pathways. The red diamond represents the drug, the blue circles represent overlapping genes, and the green circles represent pathways, p < 0.05.

To further explore the mechanism related to RA and vitamin D, GO analysis was performed, as shown in Figure 5 (Supplementary Table S5). The GO analysis involved cell proliferation, immune response, and apoptotic process, which play an essential role in vitamin D against RA. These processes occur in membrane rafts, endoplasmic reticulum, secretory granules, etc. They have functions such as molecular transducer activity, haem binding, and chemokine binding. We performed the KEGG analysis to explore which pathways are enriched for overlapping genes. Then, antifolate resistance, osteoclast differentiation and the NF-κB signalling pathway were found in the KEGG analysis (Figure 6), and they may be new pathways for vitamin D to resist RA. Among them, the top 10 pathways are as follows (Table 2). The vitamin D–target–pathway diagram of the top 10 pathways was constructed using Cytoscape software (Figure 7).

**TABLE 2:** The top 10 enriched KEGG pathway.

ID.	Description	Gene set size	Enrichment ratio	*p*-value	FDR
hsa01523	Antifolate resistance	31	35.343	3.44E-15	1.12E-12
hsa04380	Osteoclast differentiation	128	9.338	3.90E-09	6.35E-07
hsa00591	Linoleic acid metabolism	29	24.042	1.00E-08	1.09E-06
hsa00983	Drug Metabolism	79	11.347	7.34E-08	5.98E-06
hsa05204	Chemical carcinogenesis	82	10.932	1.02E-07	6.65E-06
hsa05152	Tuberculosis	179	6.677	1.70E-07	8.09E-06
hsa00590	Arachidonic acid metabolism	63	12.648	1.74E-07	8.09E-06
hsa05222	Small cell lung cancer	92	9.744	2.79E-07	1.14E-05
hsa05140	Leishmaniasis	74	10.768	6.19E-07	2.24E-05
hsa05418	Fluid shear stress and atherosclerosis	138	7.218	9.82E-07	3.20E-05

### Molecular docking technology for predicting the binding ability of vitamin D active ingredients to potential targets

**FIGURE 8:**
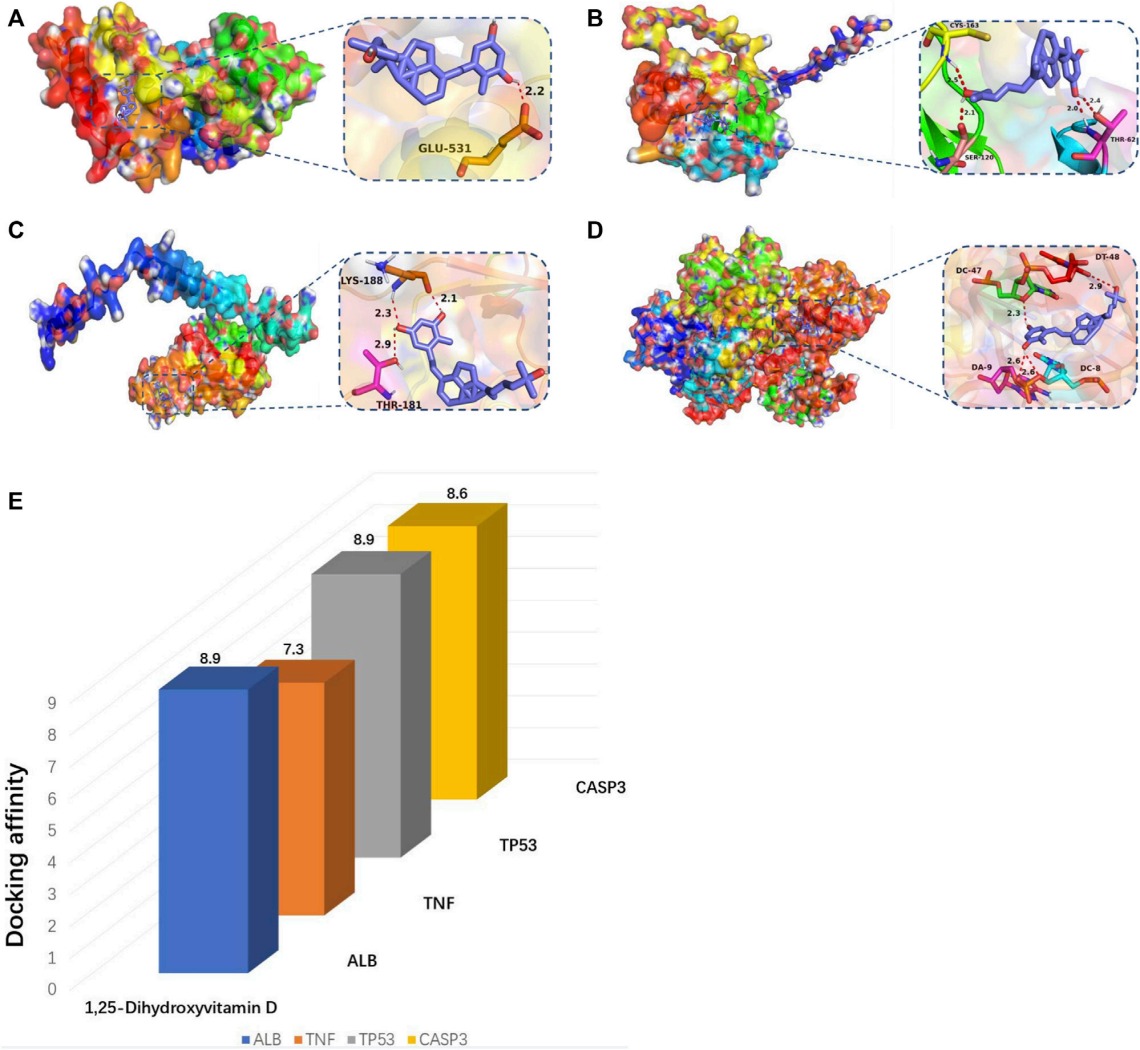
(A–E) Molecular docking map of key genes:**(A)** ALB and 1,25-Dihydroxyvitamin D (-8.9 kcal/mol); **(B)** CASP3 and 1,25-Dihydroxyvitamin D (−8.6 kcal/mol); **(C)** TNF and 1,25-Dihydroxyvitamin D (−7.3 kcal/mol); **(D)** TP53 and 1,25-Dihydroxyvitamin D (−8.9 kcal/mol).**(E)** Bar diagram of molecular docking binding energy.

According to molecular docking technology, the binding ability to the key targets TP53, ALB, CASP3, and TNF was predicted (Table 3). It is generally believed that binding energy less than −4.25 kcal/mol indicates that the ligand has a specific binding activity with the receptor, and binding energy less than −5.0 kcal/mol indicates better binding activity. Binding energy less than −7.0 kcal/mol indicates robust binding activity (Hsin et al., 2013). Through molecular docking, we found that ALB, TNF, TP53, and CASP3 have an excellent binding ability to the active ingredients of vitamin D (Figure 8). PyMOL software was used to visualise the docking results of components with solid binding activity (binding energy≤-7 kcal/mol) and the target site, as shown in Figure 8.

**TABLE 3:** The top four molecular docking with high binding energies.

Target	Docking score (kcal/mol)
ALB	−8.9
TNF	−7.3
TP53	−8.9
CASP3	−8.6

## Discussion

It is reported that RA is a common inflammatory arthritis with an increasing prevalence in many countries. (Venetsanopoulou et al., 2022). However, treatment options for many of these diseases remain inadequate for most patients, and progress in developing new treatments has been slow. This lack of progress is attributed mainly to insufficient understanding of the complex mechanisms driving pathogenesis (Cao et al., 2022; Zheng et al., 2022).

Some clinical evidence suggests that vitamin D is a critical factor in RA (Cardoso et al., 2022). RA is associated with genetic factors, autoimmunity, and inflammation (Szekanecz et al., 2021; Zhao et al., 2021). According to this research, 76 overlapping genes are essential for the vitamin D treatment of RA. ALB, TNF, CASP3, and TP53 may be critical genes among them.

As early as 2014, a research experiment on 76 RA patients showed for the first time that elevated N-homocysteine albumin in RA patients could lead to enhanced autoimmune responses, and it can be used as an indicator for clinical assessment of the severity of RA (NowAkowsk-PlAz et al., 2014). Experiments in 110 RA patients and 100 healthy individuals showed that lower concentrations of serum Alb were independently associated with the index of disease activity (Sahebari et al., 2016). Clinically, the albumin-to-fibrinogen ratio (AFR) and the C-reactive protein-to-albumin ratio (CAR) have served as inflammatory markers. AFR concentrations are decreased in RA patients, whereas CAR concentrations are increased. They can be used as potential indicators to determine inflammation in RA (He et al., 2020), and other clinical evidence suggests that patients with RA have increased ALB-dNLR scores. This suggests that the ALB-dNLR score may be a marker for assessing disease activity in RA patients (Chen et al., 2019).Additionally, methotrexate is the first-line treatment for patients with RA. Some studies show that ALB and TP53 have been identified as hub genes in the PPI network between methotrexate and RA (Bergström et al., 2018). Some animal models show that liver damage caused by MTX can cause changes in serum albumin levels, suggesting that albumin levels may indicate adverse drug reactions in RA (Hend, 2015). An important feature of the RA joint is the proliferative destruction of vascular membrane tissue regulated by activated fibroblast-like synoviocytes (FLS). The level of TP53 transcription in FLS treated with MTX increased (Wang et al., 2021).

Tumour necrosis factor (TNF)-targeting drugs have been shown to exert high effectiveness for RA, indicating the key importance of this cytokine in this disease (Wysocki and Paradowska-Gorycka, 2022). With advances in identifying immune pathogenesis in RA, TNF-α inhibitors are widely used in RA with varying success (Millier et al., 2022). In addition, TNF inhibitors have been introduced into the clinical treatment of rheumatoid arthritis (RA) for approximately 20 years. During this period, studies have shown that the TNF superfamily (TNFSF) plays a vital role in many inflammatory and autoimmune diseases. It can stimulate lymphocyte activation, increase lymphocyte survival and function or induce cell death (Locksley et al., 2001; Croft, 2003; Croft, 2009; Croft et al., 2012). In addition, TNFSF cytokines can also drive inflammatory responses (Croft et al., 2012).

Caspase-3 is a member of the cysteine protease family that cleaves its substrates mainly after aspartate residues. It plays an irreplaceable role in apoptosis and related diseases. Therefore, the detection of caspase-3 is of great significance for apoptosis imaging and the evaluation of early tumor treatment and other diseases (Lei et al., 2022; Mahshid et al., 2022).RA is alleviated by inhibiting TNF-α-induced caspase3/GSDME-mediated pyroptosis (Zhai et al., 2022).Caspase-3 is a key proenzyme in apoptosis (He et al., 2014; Asadi et al., 2021), and a previous study reported that osteoclast differentiation requires activation of caspase-3 (Szymczyk et al., 2006). Inhibition of NF-κB signaling in osteoclasts can induce apoptosis, associated with increased caspase-3 activity and decreased IL-6 expression (Piva et al., 2006; Piva et al., 2009). Sinomenine has been used in treating rheumatoid arthritis in China and Japan, and experimental studies have shown that it can inhibit osteoclast survival *in vitro* through caspase-3-mediated apoptosis (He et al., 2014).

**FIGURE 9:**
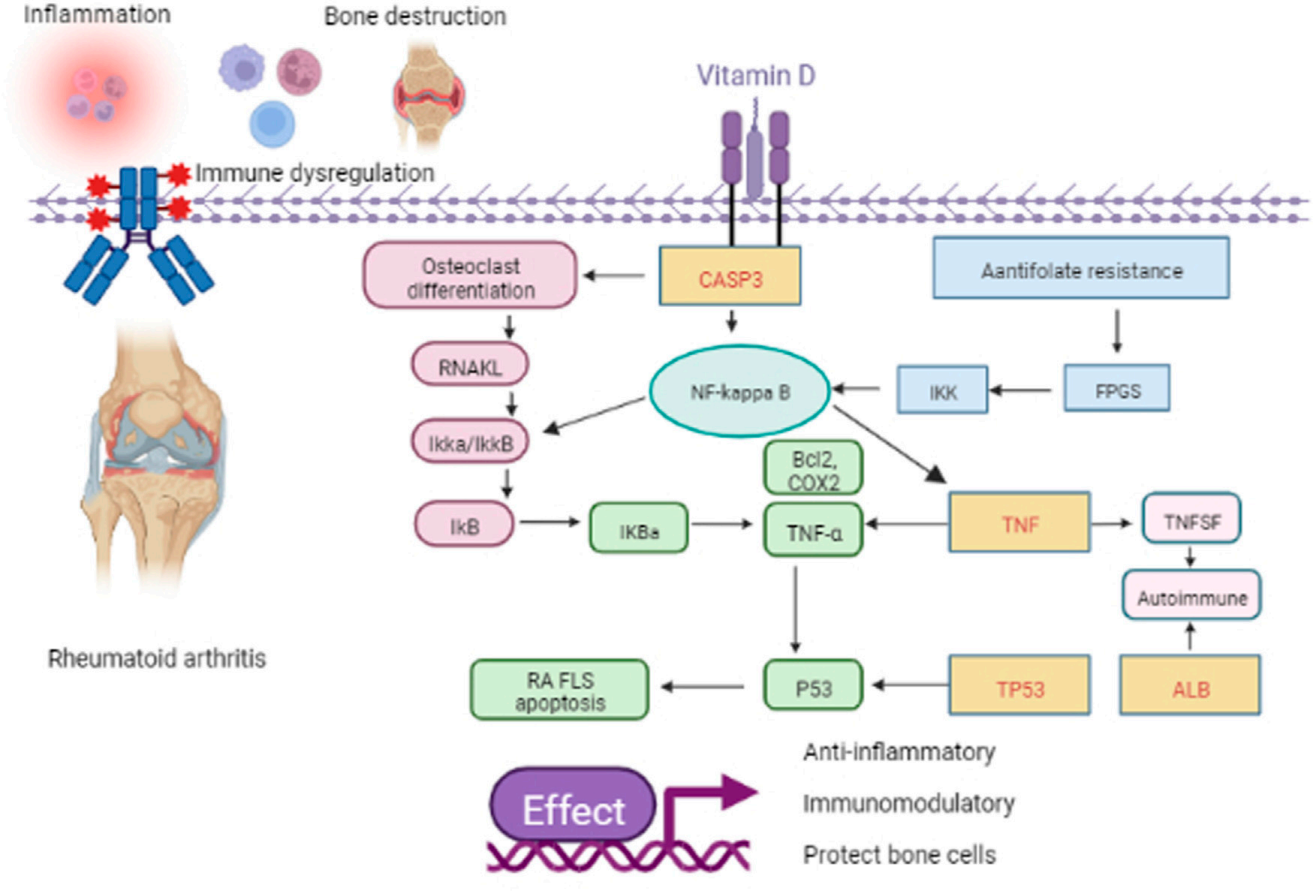
Schematic representation of the role of vitamin D in RA. The yellow rectangle with the red word represents the four key genes such as TP53, CASP3, TNF, and ALB. The pink rectangle, the blue rectangle, and the green circle represent the pathway of osteoclast differentiation, antifolate resistance, and NF-kappa B, respectively. Others represent the downstream factor.

The p53 protein, encoded by the TP53 gene, is a significant tumor suppressor (Forbes et al., 2015). Furthermore, p53 is a suppressor of inflammation and implicated in autoimmune diseases. P53 is involved in the inflammatory response by regulating inflammatory signaling pathways and inducing the expression of cytokines and matrix metalloproteinases (Gansmo et al., 2021). In addition, p53 regulates immune responses by regulating Toll-like receptor expression and innate and adaptive immune cell differentiation and maturation. P53 regulates apoptosis and proliferative processes by regulating various anti- and pro-apoptotic genes (Taghadosi et al., 2021). Impaired apoptosis of FLS causes synovial hyperplasia, facilitating the destruction of cartilage and bone in rheumatoid arthritis (RA). *In vitro,* vitamin D treated through TNF-α upregulated p53 acetylation-mediated apoptosis in MH7A cells by promoting the translocation of Sirt1 from the nucleus to the cytoplasm. This promotes FLS apoptosis and prevents RA progression (Gu et al., 2016). In this study, we focused on the role of key targets in RA. These molecules may be critical for the development of RA and are expected to be targeted for future intervention and treatment of rheumatic diseases. Wang (Wang et al., 2020) et al. showed that miR-483-3p promoted cell proliferation of FLS in RA by targeting IGF-1. It has been reported that many innate immune mechanisms produce cytokines and chemokines, such as tumor necrosis factor TNF-α, IL-6, and IL-1, involved in the pathogenesis of RA (Mormile et al., 2021). In addition, clinical studies have shown that CD14-positive monocytes from RA patients are more resistant to apoptosis, thereby promoting their persistence at the site of inflammation (Ren et al., 2019). These findings suggest cell proliferation, immune response, and apoptotic process results are related to RA. The KEGG results showed that antifolate resistance, osteoclast differentiation, and the NF-kappa B signaling pathway are essential for vitamin D in treating rheumatic disease. Daniel et al. found that folic acid antagonists have immunomodulatory effects (Fernández-Villa et al., 2019). Bone is a target organ in rheumatic disease. Inflammatory rheumatic diseases can disrupt the normal process of coupled bone remodeling, and osteoclasts can absorb bones to maintain bone integrity (Baum and Gravallese, 2016). NF-κB is an inducible transcription factor controlled by signal activation cascades (Rinkenbaugh et al., 2021). NF-κB controls many genes involved in the inflammatory immune response, cell cycle progression, apoptosis inhibition, and cell adhesion, thus promoting a chronic inflammatory response (Kizilirmak et al., 2022). In fact, in rheumatic diseases, NF-κB is activated by constituent type. Studies have shown that sinapic acid alleviates rheumatoid arthritis by reducing inflammation and oxidative stress by downregulating IκB kinase downstream of NF-κB (Okamoto, 2006; Wang et al., 2021; Wang et al., 2021). Finally, through molecular docking, we found that ALB and TP53 had the highest binding to vitamin D, followed by CASP3, and the lowest binding degree of TNF to vitamin D. The mechanism of action is shown in Figure 9.

## Conclusion

In short, through this study, we found that vitamin D treatment of RA may occur through antifolate resistance, osteoclast differentiation, and the NF-κB signaling pathway, acting on ALB, TNF, CASP3, and TP53 targets. Thus, through multiple pathways and targets, vitamin D plays a role in immune regulation and anti-inflammation. In addition, this study shows that ALB, CASP3, TNF, and TP53 may become diagnostic markers or therapeutic targets for rheumatic diseases, and further experiments are needed to explore this possibility.

## Data Availability

The original contributions presented in the study are included in the article/Supplementary Material, further inquiries can be directed to the corresponding authors.
